# Effects of individualized phase-amplitude-coupled transcranial alternating current stimulation on tinnitus and cognition

**DOI:** 10.3389/fnins.2026.1905125

**Published:** 2026-09-30

**Authors:** Youngmin Na, Nour Alsabbagh, Jayce Woojin Baek, Sunmi Ma, Hyo-Jeong Lee, Jung Hye Park, Sung Kwang Hong

**Affiliations:** 1 The Laboratory of Hearing, Balance, and Integrated Neuroscience, Hallym University College of Medicine, Chuncheon, Gangwon, Republic of Korea; 2 Department of Communication Sciences and Disorders, College of Liberal Arts and Sciences, University of Iowa, Iowa City, IA, United States; 3 Department of Otorhinolaryngology-Head and Neck Surgery, Hallym University College of Medicine, Anyang, Gyeonggi, Republic of Korea

**Keywords:** brain stimulation, cognition, cross-frequency coupling, tACS, tinnitus

## Abstract

**Introduction:**

Chronic tinnitus is a prevalent and disabling condition that is frequently accompanied by domain-specific cognitive difficulties. Abnormal neural oscillations and disrupted cross-frequency coupling (CFC) have been implicated in its pathophysiology. This study investigated whether individualized phase-amplitude-coupled transcranial alternating current stimulation (tACS), tailored to each participant’s individual alpha frequency (IAF), was associated with changes in tinnitus symptoms, cognition, and neurophysiological measures.

**Methods:**

In this prospective single-arm interventional study, 25 adults with chronic tinnitus (>6 months) were enrolled, and 21 completed the protocol. Participants received five sessions of individualized IAF-based phase-amplitude-coupled tACS at 2 mA for 20 min per session. Tinnitus outcomes included the Tinnitus Handicap Inventory (THI) and visual analog scale ratings of loudness (VAS-L) and annoyance (VAS-A). Cognitive performance was assessed using the Montreal Cognitive Assessment, Trail Making Tests A and B, Digit Span Tests forward and backward (DST-F/B), and Symbol Digit Modalities Test (SDMT). Electroencephalography-based spectral and CFC analyses were conducted in an artifact-free subgroup of 10 participants.

**Results:**

THI scores decreased significantly over the course of the intervention, whereas VAS-L and VAS-A showed no significant group-level changes. Cognitive performance improved across global cognition, processing speed, executive function, and working memory. Repeated-measures analyses demonstrated significant session effects for DST-F, DST-B, and SDMT. DST-B also showed a significant overall pre- to post-stimulation effect, whereas this effect was marginal for DST-F and absent for SDMT; no significant tACS × session interactions were observed. CFC analyses showed no significant pre- to post-stimulation changes after correction for multiple comparisons.

**Conclusion:**

Repeated individualized IAF-based phase-amplitude-coupled tACS was associated with reduced tinnitus-related functional impact and improvements in several cognitive measures, whereas subjective loudness and annoyance did not change significantly at the group level. No robust group-level CFC changes were detected. Given the single-arm design and absence of sham control, these findings are preliminary and do not establish treatment-specific or causal effects. Larger randomized sham-controlled studies are needed to determine efficacy and clarify underlying neurophysiological mechanisms.

## Introduction

Tinnitus, the phantom perception of sound without external stimuli, is believed to result from maladaptive neuroplastic changes in the central auditory system following peripheral auditory deafferentation (Shore et al., 2016). Electroencephalography (EEG) studies have provided neurodynamic evidence supporting this concept. According to the thalamocortical dysrhythmia model, auditory damage induces aberrant low-frequency theta oscillations that modulate high-frequency gamma activity within the thalamocortical network, thereby disrupting normal lateral inhibition and generating the tinnitus percept (De Ridder et al., 2015). Consistent with this framework, our previous studies demonstrated abnormal low-frequency oscillatory activity, particularly in the theta and alpha bands, involving auditory and frontal cortical regions in patients with tinnitus (Hong et al., 2016; Ahn et al., 2017).

These oscillatory abnormalities provide a rationale for neuromodulatory approaches aimed at modifying dysfunctional neural activity. Transcranial electrical stimulation (tES) has therefore emerged as a potential noninvasive treatment for tinnitus, with previous studies reporting reductions in tinnitus loudness and annoyance (Pal et al., 2015; Shekhawat et al., 2016; Shekhawat and Vanneste, 2018). However, therapeutic responses have been inconsistent, possibly reflecting differences in stimulation frequency, electrode montage, target regions, and treatment schedules. Such variability highlights the need for stimulation strategies that are more closely tailored to individual neurophysiological characteristics.

One potential target for such individualized neuromodulation is cross-frequency coupling (CFC), a mechanism through which neural oscillations at different frequencies interact to coordinate information processing across spatial and temporal scales. High-frequency oscillations are generally associated with local cortical processing, whereas lower-frequency rhythms are thought to facilitate communication across distributed neural networks. Phase–amplitude coupling (PAC) between these frequency ranges has been implicated in sensory processing, attention, and cognition. In our previous work, CFC between frontal and central regions was reduced in patients with tinnitus compared with healthy controls (Ahn et al., 2017). We therefore hypothesized that neuromodulation designed to emulate physiologically relevant cross-frequency interactions might influence abnormal oscillatory dynamics associated with tinnitus.

To investigate this possibility, we developed an individualized transcranial alternating current stimulation (tACS) paradigm in which the phase of each participant’s individual alpha frequency (IAF) was coupled to the amplitude of a gamma-frequency oscillation. This individualized phase–amplitude–coupled approach was intended to engage oscillatory interactions potentially relevant to tinnitus-related neural dysfunction. Importantly, tinnitus is not only an auditory perceptual disorder but is also frequently associated with domain-specific cognitive difficulties, particularly in attention, executive function, working memory, and processing speed (Rossiter et al., 2006; Tegg-Quinn et al., 2016; Clarke et al., 2020). These cognitive difficulties are likely multifactorial. The persistent tinnitus percept may compete with other cognitive demands for limited attentional resources, while hearing loss, which commonly accompanies tinnitus, may independently contribute to poorer cognitive performance. Thus, cognitive dysfunction in patients with tinnitus may reflect the combined influence of tinnitus-related attentional burden, hearing-related factors, and broader alterations in neural network function. Nevertheless, even when cognitive difficulties arise in part from hearing loss or other tinnitus-associated factors (Loughrey et al., 2018; Chu et al., 2020; Hamza and Zeng, 2021), modulation of dysfunctional oscillatory networks may still have the potential to support cognitive performance. This possibility provided an additional rationale for evaluating cognitive outcomes alongside tinnitus-related symptoms.

Therefore, in this prospective pilot study, we investigated whether repeated sessions of individualized IAF-based phase–amplitude–coupled tACS were associated with changes in tinnitus-related symptoms and cognitive performance in patients with chronic tinnitus. We additionally examined EEG-derived spectral and CFC measures to explore whether behavioral changes were accompanied by corresponding neurophysiological modulation.

## Methods

### Participants

A total of 25 patients (18 men and 7 women; mean age 45.5 years; range 21–60 years) with tinnitus were recruited from the Department of Otorhinolaryngology-Head and Neck Surgery at Hallym University Sacred Heart Hospital. All participants were right-handed and had experienced tinnitus for at least 6 months. The exclusion criteria were: (1) pulsatile tinnitus, (2) history of dizziness, (3) exposure to ototoxic medications (e.g., chemotherapeutic agents), and (4) neurological or psychiatric disorders. Among all 25 enrolled participants, baseline Tinnitus Handicap Inventory (THI) and Visual Analog Scale for loudness (VAS-L) scores were 49.17 ± 23.52 and 5.17 ± 2.18, respectively. All participants underwent pure-tone audiometry (PTA) at 0.5, 1, 2, 3, 8, and 12 kHz. The pure-tone average was calculated using thresholds at 0.5, 1, 2, and 3 kHz in the ear ipsilateral to the reported tinnitus. Additionally, tinnitus pitch and loudness matching tests were performed to determine individual tinnitus characteristics.

This study was approved by the Institutional Review Board of Hallym University Sacred Heart Hospital (IRB No. 2019-11-035-001), and written informed consent was obtained from all participants before enrollment. Tolerability and adverse events were monitored throughout the study through open-ended verbal inquiry conducted by the study staff at the end of each stimulation session, supplemented by spontaneous reporting. Participants were encouraged to report any discomfort or unusual sensations at any time during the study. Of the 25 enrolled participants, four discontinued the protocol; all withdrew for personal reasons unrelated to adverse events or study procedures (one after Session 1, one after Session 3, and two after Session 4).

### Development of phase-amplitude-coupled electrical stimulation

To generate frequency-coupled tACS, a 5-min eyes-closed resting-state EEG recording was obtained before tACS in each session to determine the IAF. Following subject-specific preprocessing, the power spectral densities at F4, T7, and T8 were visually inspected to identify a distinct peak within the 6–13 Hz frequency range. The 6–13 Hz interval was used as a prespecified peak-detection window rather than as a redefinition of the conventional alpha band. Its lower bound was chosen to avoid excluding participants whose dominant resting peak was slower than 8 Hz, while the upper bound allowed interindividual variability around the conventional alpha range; this approach is consistent with prior work emphasizing substantial individual variability in alpha- and theta-range peak frequencies (Klimesch, 1999). Related electrophysiological work has also used extended lower frequency bounds (e.g., beginning at 7 Hz) when identifying an individual alpha peak (Li et al., 2023). Visual inspection was restricted to the prespecified target channels and frequency window and was used to confirm the dominant local spectral maximum before frequency selection. When multiple peaks were present, the peak with the highest spectral density was selected as the IAF. Thus, the IAF was determined individually for each participant at each session and used as the low-frequency component of the stimulation waveform. In contrast, the gamma-frequency component (50 Hz), stimulation intensity, stimulation duration, and ramp-up and ramp-down periods were kept constant across participants and sessions. The F4 electrode position was likewise fixed, whereas the second electrode (T7 or T8) was selected according to each participant’s tinnitus laterality.

Once the session-specific IAF was determined, the stimulation waveform was generated by coupling the phase of the IAF oscillation to the amplitude of the 50-Hz gamma oscillation (Figure 1). The stimulation current was gradually ramped up to 2 mA over 10 s at the onset and ramped down over 10 s at the end of stimulation to minimize discomfort. Based on previous evidence implicating the right dorsolateral prefrontal cortex (DLPFC) as a critical hub in tinnitus pathophysiology, this region was selected as the stimulation target (Schlee et al., 2009; Schutter, 2010; Vanneste et al., 2010). For this purpose, one electrode was positioned at F4 according to the international 10–20 electrode placement system. The second electrode was placed at either T7 or T8, depending on tinnitus laterality. For instance, in patients with right-sided tinnitus, the second electrode was positioned at T8. In patients with bilateral tinnitus, the temporal electrode was placed on the side corresponding to the ear in which tinnitus was perceived as louder. Stimulation was delivered continuously for 20 min using NG Pistim electrodes (π cm^2^) in conjunction with a Starstim 32 device (Neuroelectrics, Barcelona, Spain).

**FIGURE 1:**
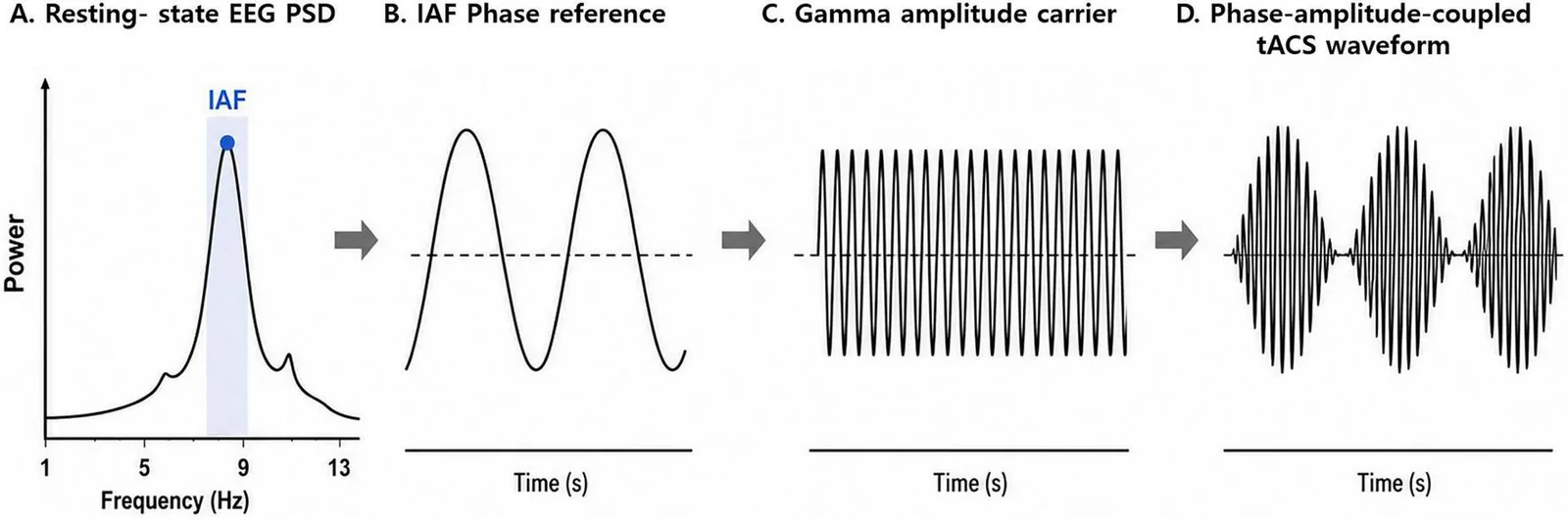
A schematic illustration of an individual alpha frequency (IAF)-based phase–amplitude-coupled stimulation waveform. **(A)** Resting-state EEG power spectral density (PSD) in the 6–13 Hz range, with the peak frequency identified as the IAF (blue dot). **(B)** Low-frequency IAF oscillation providing the phase reference for stimulation. **(C)** High-frequency gamma oscillation (50 Hz), representing the fast rhythm used as the amplitude component in the coupling scheme. **(D)** Final individualized tACS waveform representing the IAF-based phase–amplitude-coupled signal. The waveform was generated by coupling the phase of the IAF oscillation shown in **(B)** to the amplitude of the gamma oscillation shown in **(C)**, such that gamma amplitude was maximal at specific phases of the IAF cycle.

To characterize the spatial distribution of the applied electric field for the F4–T7 and F4–T8 montages, finite-element simulations were performed using SimNIBS 4 (v4.0; Technical University of Denmark, Copenhagen, Denmark) with the standard “ernie” head model and 20-mm spherical cortical regions of interest (ROIs) beneath F4, T7, and T8. The simulations demonstrated electric-field delivery to the intended target regions, with 99th-percentile electric-field magnitudes of 0.37–0.40 V/m in the right DLPFC, 0.47 V/m in the left auditory cortex for the F4–T7 montage, and 0.58 V/m in the right auditory cortex for the F4–T8 montage (Supplementary Table 1 in Supplementary file 1).

### Experimental procedure

This study aimed to evaluate the effects of cross-frequency-coupled tACS on tinnitus-related symptoms and cognitive function. The research protocol consisted of five stimulation sessions, each separated by an intersession interval of three to four days. The interval was selected to minimize potential acute carry-over effects between consecutive stimulation sessions. The experimental procedure began with a baseline evaluation of tinnitus-related symptoms and cognitive function (pre-tACS), followed by resting-state EEG recordings, administration of tACS, and post-stimulation assessments.

In the first session (S1), the participants completed a comprehensive evaluation battery before tACS. Tinnitus-related symptoms were assessed using the THI and a Visual Analog Scale (VAS) for tinnitus loudness (VAS-L) and annoyance (VAS-A). Cognitive assessments included the Montreal Cognitive Assessment (MoCA) for global cognition, the Trail Making Test (TMT), including Parts A (TMT-A) and B (TMT-B), the Digit Span Test (DST), including forward (DST-F) and backward (DST-B) conditions, and the Symbol Digit Modalities Test (SDMT). The cognitive battery was selected to target the domains most consistently implicated in tinnitus-related cognitive dysfunction: the MoCA was used as a brief global cognitive screen; the TMT-A and TMT-B assessed processing speed, cognitive flexibility, and executive function, domains repeatedly shown to be impaired in patients with tinnitus during attention-demanding tasks; the DST evaluated working memory, reflecting the hypothesized depletion of attentional resources by the tinnitus percept; and the SDMT provided an additional, repeatable measure of processing speed and attention suitable for use across all five sessions (see Supplementary File 2 for further details).

The DST and SDMT were assessed before and after tACS across all five sessions (S1–S5), yielding a total of 10 measurements each. Tinnitus-related measures (THI, VAS-L, and VAS-A) were assessed before tACS in Sessions 1–4, with the final measurement taken after tACS in Session 5, yielding a total of five measurements each. The MoCA and TMT were administered before tACS in the first session (S1) and repeated after tACS in the final session (S5) to evaluate changes from baseline to the end of the intervention (Figure 2).

**FIGURE 2:**
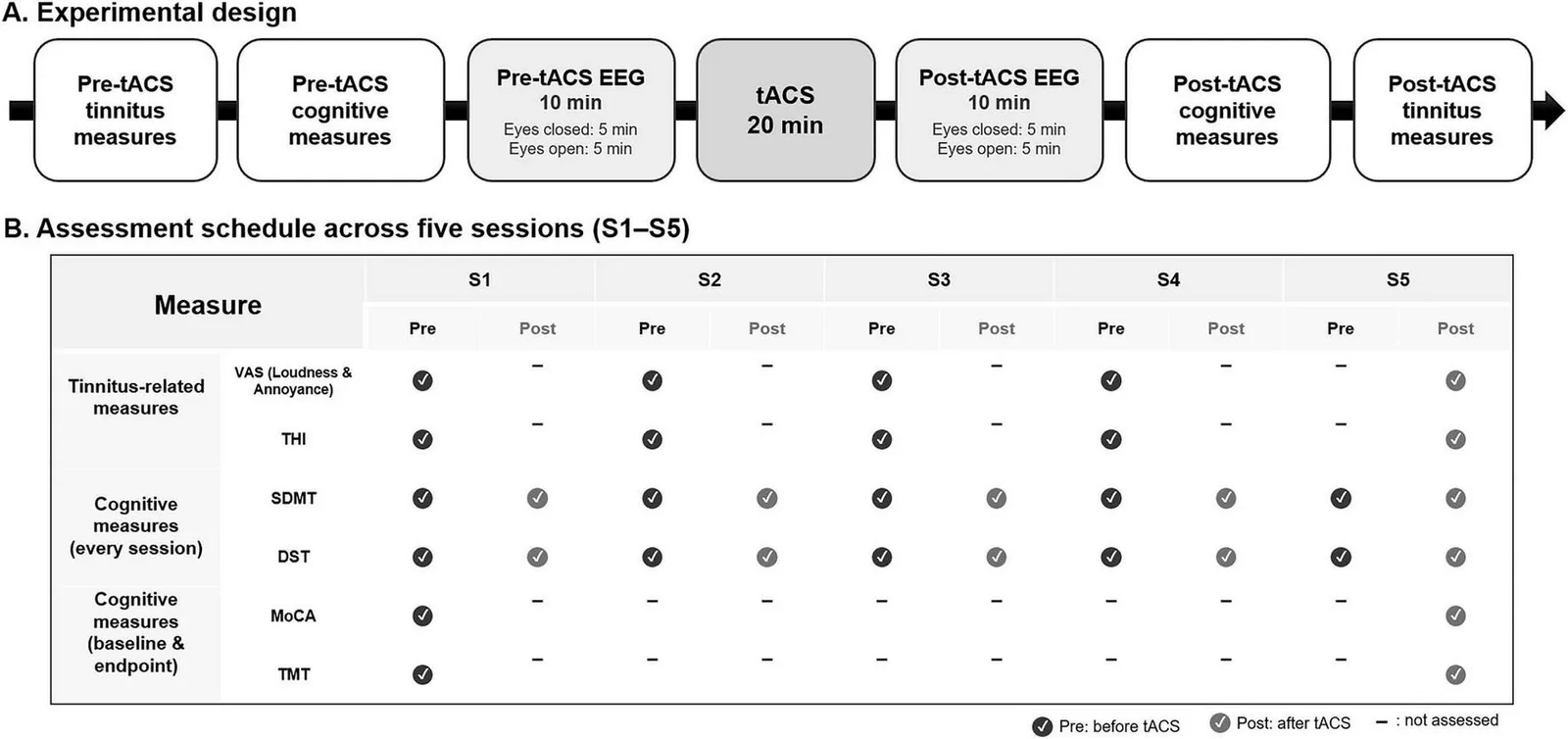
Experimental design overview. **(A)** Experimental design: Each session began with pre-tACS tinnitus-related and cognitive assessments, followed by a 10-min pre-tACS resting-state EEG recording (a 5-min eyes-closed block followed by a 5-min eyes-open block), 20 min of tACS administration, a 10-min post-tACS resting-state EEG recording (a 5-min eyes-closed block followed by a 5-min eyes-open block), and post-tACS cognitive and tinnitus-related assessments. The individual alpha frequency (IAF) was determined from the pre-tACS eyes-closed EEG recording in each session and used to generate the session-specific stimulation waveform. The EEG–tACS portion of each session lasted approximately 40 min in total. **(B)** Assessment schedule across five sessions (S1–S5): Tinnitus-related measures (Visual Analog Scale [VAS] for loudness and annoyance, and the Tinnitus Handicap Inventory [THI]) were administered before tACS in Sessions 1–4, with the final measurement taken after tACS in Session 5. The Symbol Digit Modalities Test (SDMT) and Digit Span Test (DST) were administered before and after tACS in every session (S1–S5). The Montreal Cognitive Assessment (MoCA) and Trail Making Test (TMT) were administered before tACS only in Session 1 and after tACS only in Session 5, to assess change from baseline to the end of the intervention.

#### EEG measurement and CFC calculation

Resting-state EEG was recorded for 10 min at each timepoint, consisting of two 5-min blocks with alternating eyes-open and eyes-closed conditions, both before and after tACS, yielding a total of 20 min of EEG recording per session. Combined with the 20-min tACS intervention, the entire EEG–tACS procedure lasted 40 min per session. During the eyes-open condition, participants were instructed to fixate on a cross presented at the center of the screen, whereas during the eyes-closed condition, they were asked to remain still with their eyes closed. To minimize artifacts, participants were instructed to remain awake, avoid mental rehearsal or active thinking, refrain from excessive blinking during the eyes-open condition, and minimize body movements throughout the recording.

EEG data were acquired using a Starstim 32 wireless tES–EEG system (Neuroelectrics, Barcelona, Spain), which was used for both EEG recording and tACS administration. A total of 32 electrodes were positioned according to the international 10–20 system. The Driven Right Leg (ground) and common-mode sense electrodes were placed on the mastoid. Data were sampled at 500 Hz, and EEG preprocessing was performed using the EEGLAB toolbox (Delorme and Makeig, 2004). The raw EEG signals were first filtered with a 1-Hz high-pass filter and a 60-Hz notch filter to remove slow drifts and line noise, respectively. Artifactual components, such as those related to eye blinks, muscle activity, and other non-neural sources, were identified and removed offline using independent component analysis (Makeig et al., 1997). Identified bad channels were interpolated, and EEG signals were re-referenced to the common average and segmented into 2-s epochs for subsequent PAC analysis. Epochs beginning within the first 6 s of each recording and those exceeding a voltage threshold of ± 50 μV were rejected to minimize artifact contamination. Power spectral density (PSD) was estimated using Welch’s method with a 1-s Hanning window and 50% overlap. Absolute band powers were calculated by integrating the PSD across specific frequency ranges: theta (4–8 Hz) and alpha (8–12 Hz). The Theta/Alpha Ratio (TAR) was defined as the ratio of absolute theta power to absolute alpha power:


$$T\,A\,R\,P\,o\,w\,e\,r_{\theta }/P\,o\,w\,e\,r_{\alpha }$$

averaged across the target channels (F4, T7, and T8). IAF was identified as the frequency displaying the peak spectral power within the 6–13 Hz range across the target channels during the eyes-closed resting-state condition. For the CFC analysis, PAC values were computed between the phase of low-frequency oscillations (1–12 Hz; below the gamma range) and the amplitude envelope of higher-frequency activity (up to 200 Hz). PAC estimation was performed using the PAC Toolbox, which allowed quantification of the coupling strength between these frequency bands (Onslow et al., 2011). Both frequency bands were further subdivided into 1 Hz bins to enable granular analysis. Due to strict signal-quality and artifact-free criteria, all quantitative EEG analyses, including spectral measures (TAR and IAF) and CFC, were conducted in a final cohort of 10 participants. For this artifact-free EEG cohort (n = 10), baseline (S1) resting-state parameters were as follows: the target-channel TAR was 0.92 ± 0.28, and the IAF was 9.38 ± 2.36 Hz.

### Statistical analysis

Statistical analyses were performed using SPSS version 25 software (IBM Corp., Armonk, NY, USA). Prespecified baseline-to-endpoint cognitive changes (MoCA, TMT-A, TMT-B, DST, and SDMT; Session 1 pre-tACS vs. Session 5 post-tACS) were evaluated using paired-samples t tests. Changes in tinnitus-related outcomes across sessions were assessed separately for THI, VAS-A, and VAS-L using repeated-measures analysis of variance (RM-ANOVA) with session (S1–S5) as the within-subject factor. A 2 × 5 RM-ANOVA with tACS condition (pre- and post-stimulation) and session (S1–S5) as within-subject factors was performed for DST-F, DST-B, and SDMT to examine the main effects of session and tACS condition and their interaction. Sphericity was assessed using Mauchly’s test, and Greenhouse–Geisser corrections were applied when necessary. Post hoc pairwise comparisons were adjusted using the Bonferroni correction. Statistical significance was set at α = 0.05, and descriptive statistics are reported as mean ± standard deviation (SD) unless otherwise specified.

Because *a priori* sample size calculation was not performed, a sensitivity analysis was conducted to determine the minimum detectable paired effect size given the final sample size. With 21 completers, the study had 80% power to detect a two-sided paired-samples effect of approximately Cohen’s dz = 0.64 at α = 0.05. Data distributions were evaluated using the Shapiro–Wilk test together with visual inspection of Q–Q plots. Given the robustness of paired-samples and repeated-measures procedures to moderate deviations from normality, parametric analyses were retained where appropriate. Potential outliers and influential observations were assessed using prespecified diagnostic criteria. To account for potential confounding from the broad age range, correlation analyses between age and outcome measures were performed, and analysis of covariance (ANCOVA) was conducted with age as a covariate; age was not significantly associated with the primary outcome measures, and adjustment for age did not materially alter the overall pattern of results.

All post hoc pairwise comparisons following the repeated-measures analyses were adjusted using the Bonferroni correction, and the reported *p* values represent Bonferroni-adjusted *p* values. Effect sizes for repeated-measures ANOVA/ANCOVA were reported as eta squared (η^2^).

For CFC analysis, electrode sites were selected based on established evidence: (1) auditory processing dipole sources typically exhibit the most prominent activity around the vertex (Näätänen, 1992); (2) the Fz area is widely recognized as a representative location for prefrontal top-down processing (Keeser et al., 2011); and (3) a previous study on CFC patterns in patients with tinnitus (Ahn et al., 2017) focused on CFC between phase at Fz and amplitude at Cz, as well as phase at Cz and amplitude at Fz following tACS. We specifically explored CFC involving low-frequency oscillations, including delta (1–4 Hz), theta (4–8 Hz), and alpha (8–12 Hz) phases, and their modulation of high gamma (50–200 Hz) amplitude. This research direction was chosen based on previous studies suggesting that the amplitude of high-gamma oscillations is influenced by the phase of lower-frequency oscillations (Buzsáki and Draguhn, 2004; Canolty et al., 2006). The maximum PAC values within these frequency ranges were compared using the Wilcoxon signed-rank test.

## Results

### Tinnitus-related measures

RM-ANOVA with session as the within-subject factor (comprising S1, S2, S3, S4, and S5) demonstrated a significant effect of session on THI scores [F(2.239, 44.776) = 11.118, *p* < 0.001] (Figure 3A). Post hoc pairwise comparisons (Bonferroni-corrected) revealed significant differences in the THI scores between S1 and S3 (*p* = 0.005), S1 and S4 (*p* = 0.040), S1 and S5 (*p* = 0.003), and S2 and S5 (*p* = 0.010).

**FIGURE 3:**
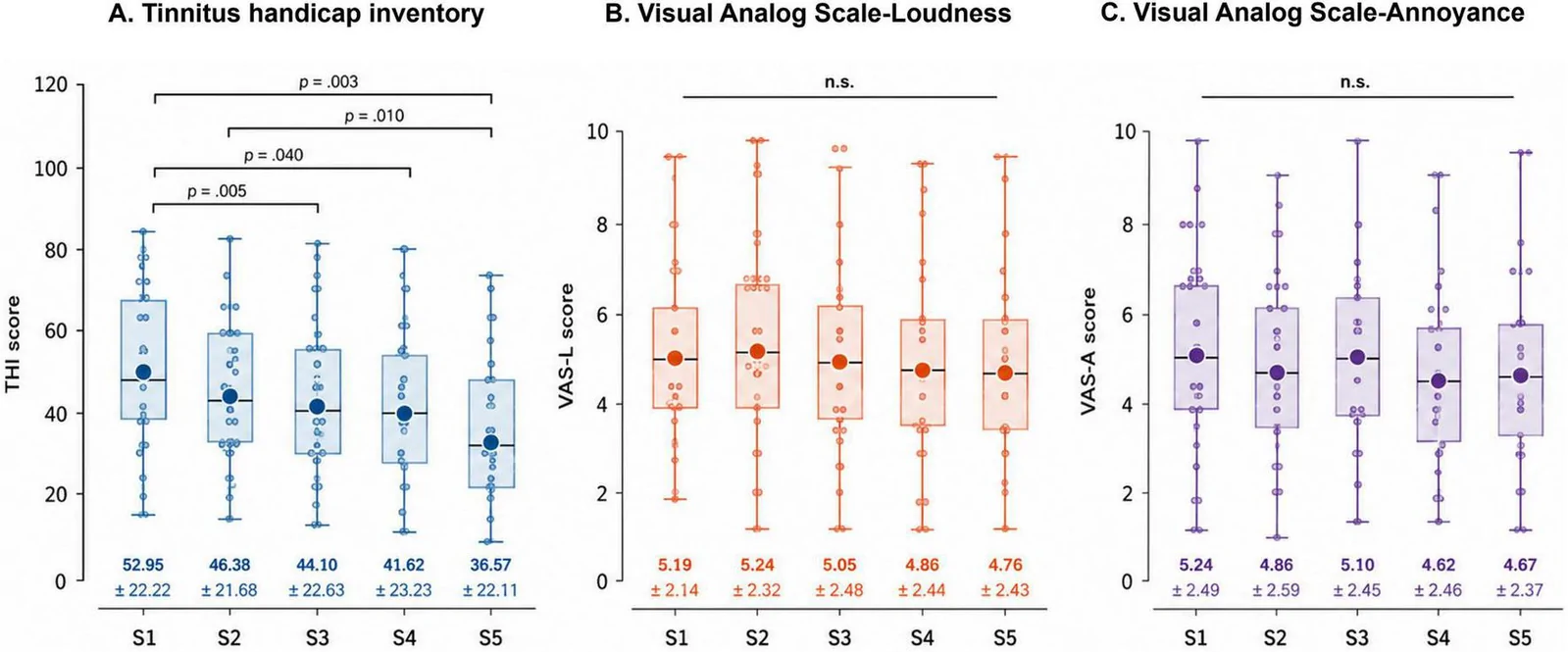
Changes in Tinnitus Handicap Inventory (THI) and Visual Analog Scale (VAS) scores across five tACS intervention sessions (S1–S5). Box plots depict the median, interquartile range, and full range of individual participant scores (open circles), with filled circles connected by lines indicating group means; mean ± SD values are reported below each session. **(A)** THI scores showed a significant main effect of session (*p* < 0.001), with significant Bonferroni-corrected pairwise differences indicated in the Figure. **(B)** VAS loudness (VAS-L) and **(C)** VAS annoyance (VAS-A) scores showed no significant session effects. Asterisks indicate statistical significance (*p* < 0.05, *p* < 0.01, *p* < 0.001).

For VAS-L and VAS-A, repeated-measures ANOVA of the variable “session” did not reveal a significant effect for either measure (VAS-L: F(2.853, 57.054) = 2.182, *p* = 0.103; VAS-A: F(2.495, 49.902) = 2.417, *p* = 0.088) (Figures 3B, C). None of the pairwise comparisons between the sessions were statistically significant for either measure.

### Cognitive measures

Paired-samples *t*-tests revealed significant improvements in cognitive scores after tACS sessions. Specifically, the MoCA score increased significantly from 26.71 ± 2.43 at baseline (S1 pre-tACS) to 28.29 ± 2.19 after the final session (S5 post-tACS; t = –4.418, *p* < 0.001), consistent with improved global cognitive performance (Figure 4A). Working memory, as assessed using the DST, also showed significant improvement. DST-F scores increased from 7.05 ± 1.28 to 7.86 ± 1.42 (t = –3.302, *p* = 0.004) (Figure 4B), and DST-B scores increased from 3.57 ± 0.51 to 5.10 ± 1.41 (t = –5.261, *p* < 0.001) (Figure 4C). To further examine session effects, 2 × 5 repeated-measures ANOVA was conducted separately for DST-F and DST-B. For DST-F (n = 21), there was a significant main effect of session [F(2.564, 51.278) = 15.498, *p* < 0.001, η^2^ = 0.061], a marginal main effect of tACS [F(1, 20) = 4.213, p = 0.053], and no significant interaction (*p* = 0.845). Bonferroni-adjusted comparisons revealed significant differences between S1 and S2 (*p* = 0.002), S1 and S3 (*p* = 0.016), S1 and S4 (*p* < 0.001), and S1 and S5 (*p* < 0.001). For DST-B (n = 19; two participants were excluded from this analysis owing to incomplete responses at the lowest digit-span level), the ANOVA indicated a significant main effect of session [F(2.839, 51.094) = 9.710, *p* < 0.001, η^2^ = 0.111] and a significant main effect of tACS [F(1, 18) = 6.429, *p* = 0.021], with no significant interaction (*p* = 0.673). Pairwise comparisons showed significant improvements from S1 to S2 (*p* = 0.025), S1 to S4 (*p* = 0.021), S1 to S5 (*p* = 0.002), and S2 to S5 (*p* = 0.033). For TMT, significant reductions in completion time were observed after S5. TMT-A performance improved from 35.45 ± 12.69 s to 28.32 ± 9.10 s (t = 3.192, *p* = 0.005) (Figure 4D), and TMT-B scores decreased from 79.66 ± 39.74 s to 67.64 ± 35.37 s (t = 2.551, *p* = 0.019) (Figure 4E). These reductions are consistent with improved processing speed, cognitive flexibility, and visuomotor coordination. Similarly, performance on the SDMT improved significantly from baseline (48.38 ± 12.19) to the final session (58.62 ± 12.44; t = –4.502, *p* < 0.001) (Figure 4F). A 2 × 5 repeated-measures ANOVA confirmed a significant main effect of session [F(3.308, 66.153) = 28.245, *p* < 0.001, η^2^ = 0.079], but no significant main effect of tACS (*p* = 0.826) or interaction (*p* = 0.831). Bonferroni-corrected comparisons indicated significant improvements between S1 and S2 (*p* < 0.001), S1 and S3 (*p* < 0.001), S1 and S4 (*p* < 0.001), S1 and S5 (*p* < 0.001), S2 and S4 (*p* = 0.024), and S2 and S5 (*p* < 0.001). Across the three working-memory and processing-speed measures (DST-F, DST-B, and SDMT), a significant between-session improvement was consistently observed. Notably, DST-B showed a significant main effect of tACS (pre- vs. post-stimulation), whereas DST-F showed only a marginal tACS effect and SDMT showed none; no measure showed a significant tACS × session interaction (Supplementary Figure 1).

**FIGURE 4:**
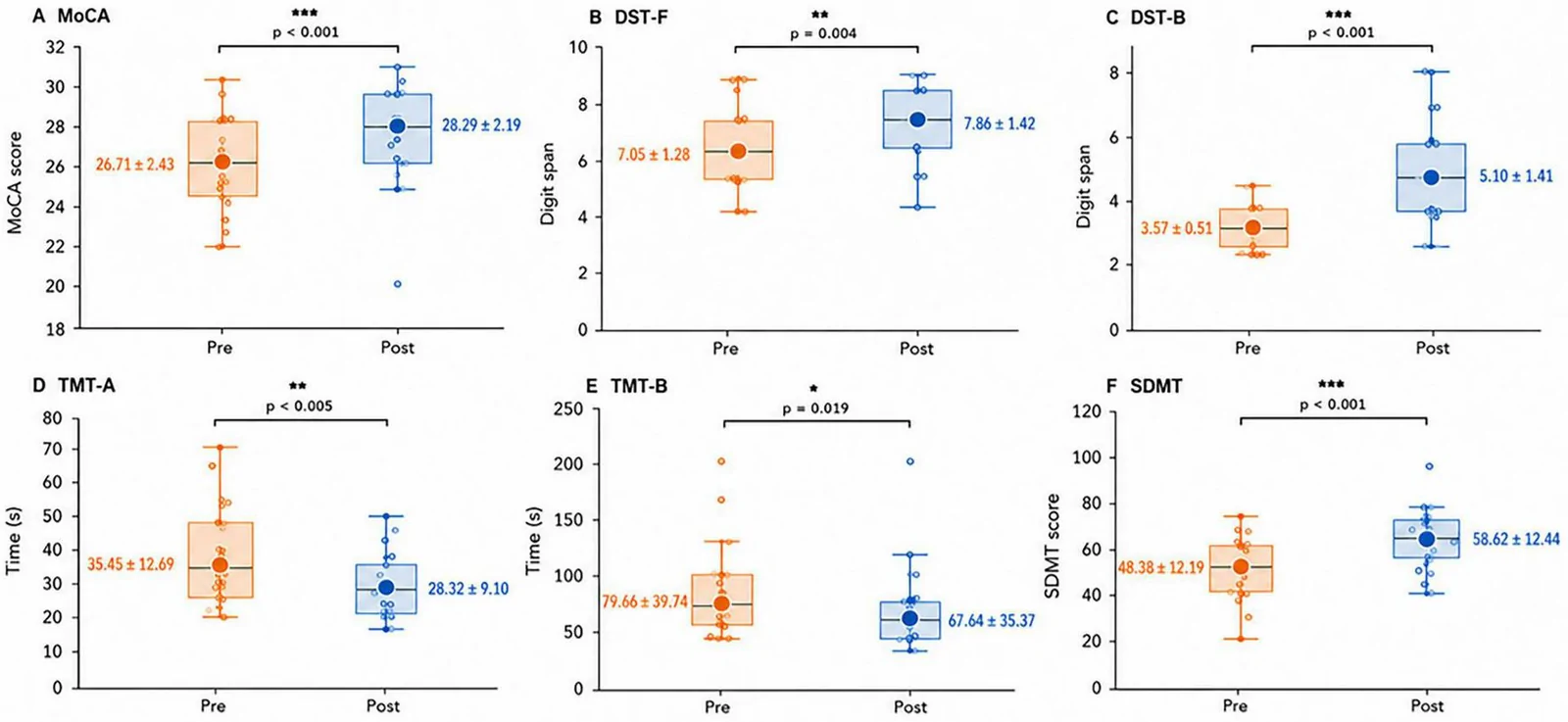
Changes in cognitive performance from baseline to the end of the intervention. Box plots depict individual participant scores (open circles) and group mean ± SD values for pre-tACS (S1) and post-tACS (S5) measurements. **(A)** MoCA, **(B)** DST-F, **(C)** DST-B, **(D)** TMT-A, **(E)** TMT-B, and **(F)** SDMT. Significant baseline-to-endpoint differences were observed for all measures. Lower completion times indicate better performance for TMT-A and TMT-B, whereas higher scores indicate better performance for the other measures. Asterisks indicate statistical significance (*p < 0.05, **p < 0.01, ***p < 0.001).

### Electrophysiological correlates of clinical and cognitive changes

To investigate whether neurophysiological modulation was associated with clinical and cognitive changes, we examined the associations between changes in the Theta/Alpha Ratio (ΔTAR) and changes in clinical outcome measures using non-parametric rank-order correlation analyses. A statistically significant negative correlation was observed between ΔTAR and changes in subjective tinnitus loudness (ΔVAS-L: Spearman’s ρ = –0.657, *p* = 0.0392). Conversely, no significant correlations were found with other clinical indices, including THI, VAS-A, MoCA, and TMT scores (p > 0.05). In addition, CFC analyses did not reveal significant pre- to post-stimulation differences following False discovery rate (FDR) correction. In both eyes-open and eyes-closed conditions, uncorrected –log10(*p*) maps suggested localized trends, but none survived FDR adjustment (all FDR-corrected *p* > 0.05; Supplementary Figures 2 and 3). Figure 5 presents a comparison between the first session pre-tACS and the last session post-tACS for both eyes-open and eyes-closed conditions across phase and amplitude frequencies. Although visual inspection suggested a trend toward enhanced alpha–gamma coupling after repeated stimulation, these differences were not statistically significant after correction for multiple comparisons using the FDR method (FDR-corrected *p* > 0.05).

**FIGURE 5:**
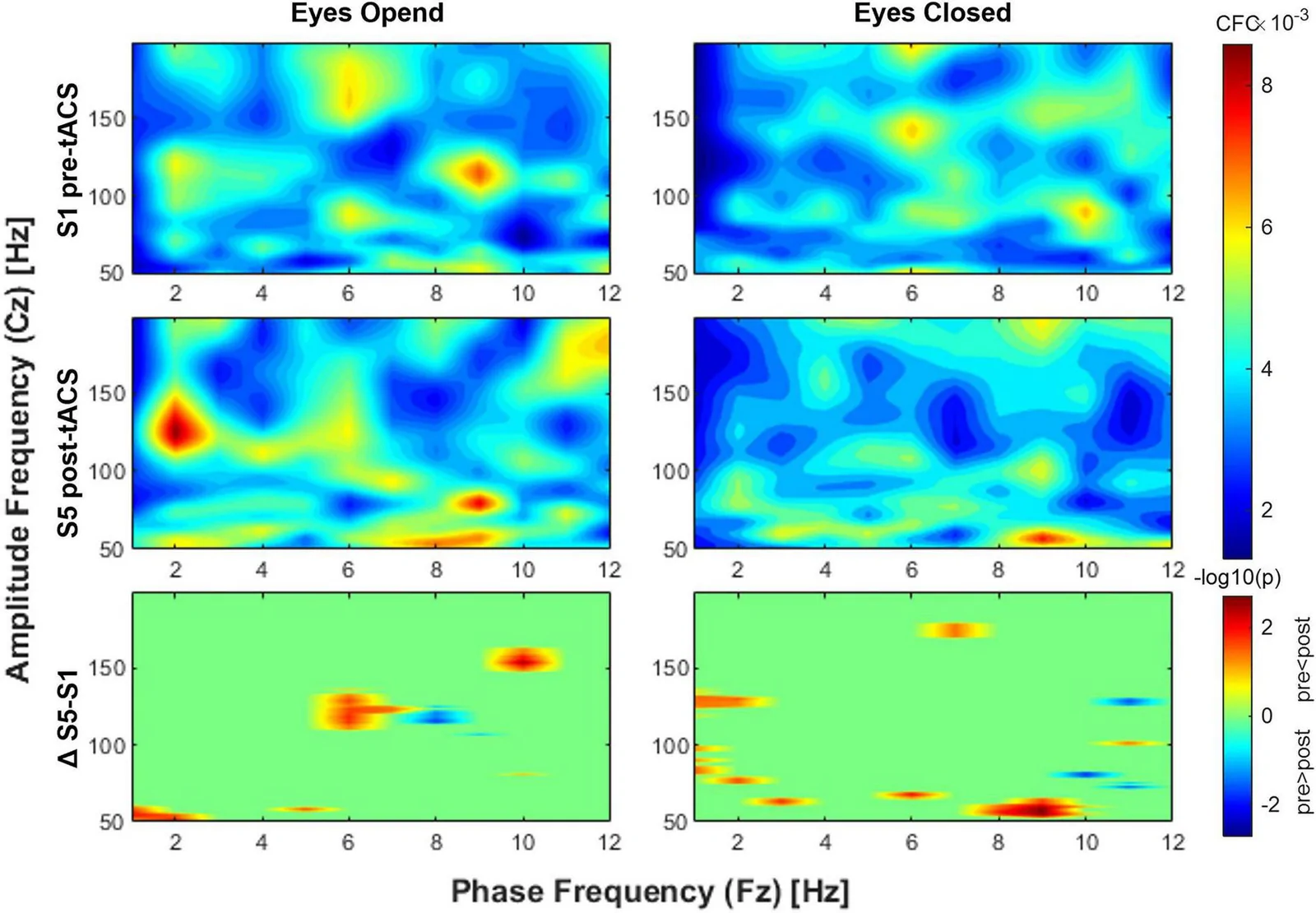
Comparison of cross-frequency coupling (CFC) between the first pre-tACS session and the final post-tACS session in eyes-open and eyes-closed conditions. Left panels: Eyes-open condition. Right panels: Eyes-closed condition. Top row: CFC comodulograms from the first pre-tACS session (Session 1). Middle row: Corresponding comodulograms from the final post-tACS session (Session 5). Phase frequencies are shown on the x-axis (1–12 Hz) and amplitude frequencies on the y-axis (50–200 Hz). The color scale represents the strength of phase–amplitude coupling (CFC × 10^−3^). Bottom row: Uncorrected –log10(*p*) values for comparisons between Session 1 (pre-tACS) and Session 5 (post-tACS). Warm colors (red) indicate greater CFC in Session 5 (post-tACS), and cool colors (blue) indicate greater CFC in Session 1 (pre-tACS).

## Discussion

Extensive research has investigated neural communication across brain regions and neural oscillations at different frequencies in patients with tinnitus. Within the tinnitus network, which includes the DLPFC, anterior cingulate cortex, hippocampus, parahippocampus, and insula, impaired modulation of neural oscillations across frequency bands has been proposed as a potential mechanism underlying tinnitus perception (Schlee et al., 2009; Vanneste et al., 2010; De Ridder et al., 2015). Consistent with this framework, our previous study demonstrated that PAC between delta- and gamma-band oscillations in frontal and central regions was absent in patients with tinnitus compared with healthy controls (Ahn et al., 2017).

Within a broader neurophysiological framework, high-frequency oscillations are thought to reflect relatively local cortical information processing, whereas low-frequency oscillations may facilitate long-range communication across cortical networks (Canolty et al., 2006; Osipova et al., 2008). CFC may therefore provide a mechanism through which neural activity occurring at different temporal scales is coordinated. CFC has been implicated in diverse cognitive and sensory processes, including working memory, attention, vision, olfaction, and auditory processing (Palva and Palva, 2007; Sauseng et al., 2010; Papadaniil et al., 2015; Siebenhühner et al., 2016). Abnormal CFC has also been reported in neurological and psychiatric disorders, including schizophrenia (Allen et al., 2011), obsessive-compulsive disorder (Bahramisharif et al., 2016), and Alzheimer’s disease (Goutagny et al., 2013). These observations provided the rationale for investigating whether individually tailored CFC-based tACS could be associated with changes in both tinnitus symptoms and cognitive function.

In the present study, CFC-based tACS tailored to each participant’s IAF was associated with a progressive reduction in tinnitus-related functional impact. THI scores showed a significant main effect of session, and post hoc comparisons indicated significant reductions between the first and third sessions, the first and fourth sessions, the first and final sessions, and the second and final sessions. Together, these findings indicate that tinnitus-related functional impact decreased progressively over the course of the intervention rather than changing abruptly at a single session. However, because no sham or control condition was included, this improvement cannot be attributed specifically to tACS and may also have been influenced by expectation, spontaneous symptom fluctuation, regression to the mean, or other time-related factors.

In contrast to the clear reduction in THI scores, subjective ratings of tinnitus loudness (VAS-L) and annoyance (VAS-A) did not show statistically robust group-level changes. Repeated-measures analyses revealed no significant session effect for either measure. This dissociation between THI and the VAS measures may reflect the multidimensional nature of tinnitus. Whereas the THI captures broader functional, emotional, and social consequences of tinnitus, single-item VAS ratings represent momentary perceptual or affective experiences that may fluctuate according to attention, context, and other transient factors. The modest sample size and limited sensitivity of single-item scales may also have reduced the ability to detect smaller changes. Thus, the present findings suggest that changes in tinnitus-related functional impact were more consistently observed than changes in momentary perceived loudness or annoyance, although confirmation of a treatment-specific effect will require a larger sham-controlled study.

Beyond tinnitus-related outcomes, cognitive performance also improved over the course of the intervention. MoCA scores increased, TMT-A and TMT-B completion times decreased, and DST-F, DST-B, and SDMT performance improved. Across DST-F, DST-B, and SDMT, significant main effects of session were consistently observed, indicating that cognitive performance changed over repeated sessions. Importantly, however, the pattern of within-session change differed across measures. DST-B showed a significant main effect of tACS (pre- vs. post-stimulation), whereas the corresponding effect was marginal for DST-F (*p* = 0.053) and absent for SDMT. None of these measures showed a significant tACS × session interaction.

Accordingly, the overall pattern primarily indicates progressive cognitive improvement across repeated sessions rather than evidence that the magnitude of the acute pre- to post-stimulation response increased with successive sessions. The additional significant pre- to post-stimulation effect observed for DST-B nevertheless raises the possibility that a within-session component may have contributed specifically to improvement in backward digit span. This distinguishes DST-B from DST-F and SDMT, for which improvement was predominantly expressed as a session-related change. However, even for DST-B, the lack of a sham condition prevents the pre- to post-stimulation difference from being attributed specifically to tACS. Repeated testing, learning, expectancy, or other time-dependent factors may have contributed to the observed changes. Furthermore, although the intersession interval was intended to minimize immediate carry-over effects, it cannot exclude the possibility of longer-lasting effects from previous stimulation sessions. Thus, the cognitive findings should be interpreted as improvements occurring over the course of repeated tACS sessions, with a possible additional within-session component for DST-B, rather than as evidence of either an established acute effect or a cumulative causal effect of stimulation.

To examine whether the observed clinical and cognitive changes were accompanied by corresponding neurophysiological changes, we further explored associations between spectral EEG modulation and behavioral outcomes. A greater reduction in ΔTAR was significantly correlated with a greater reduction in subjective tinnitus loudness (ΔVAS-L). Notably, this association was observed despite the absence of a significant group-level change in VAS-L. This suggests that the magnitude of individual change in perceived loudness, rather than a uniform directional change across the group, covaried with the degree of spectral EEG modulation across participants. Although exploratory, this finding is consistent with a possible relationship between oscillatory activity and changes in the perceptual component of tinnitus.

However, this association should be interpreted cautiously. The present study was uncontrolled and the analysis was correlational; therefore, it cannot establish that the observed spectral changes were induced by tACS or that they causally mediated tinnitus improvement. Furthermore, other clinical and cognitive measures were not significantly associated with ΔTAR in the EEG subgroup (*n* = 10). In particular, ΔTAR was associated with changes in subjective tinnitus loudness but not with any cognitive measure. Thus, the observed cognitive improvements cannot be attributed to this specific neurophysiological pathway on the basis of the available data. The finding also represented a single significant association among several exploratory comparisons within a small EEG subsample and therefore requires replication in a larger cohort with prespecified brain–behavior analyses.

The present findings may nevertheless be considered within the theoretical framework of thalamocortical dysrhythmia (TCD), which has been proposed to account for abnormal oscillatory interactions in tinnitus (De Ridder et al., 2015). From this perspective, alpha-gamma CFC-based stimulation could potentially influence the abnormal relationship between low-frequency activity and gamma-band activity within auditory networks, possibly affecting alpha-related inhibitory control over auditory cortical activity (Keeser et al., 2011; Ahn et al., 2017). Modulation of frontoparietal oscillatory networks could also theoretically influence cognitive processes such as attention, working memory, and executive control (Palva and Palva, 2007; Osipova et al., 2008). However, these mechanisms remain hypothetical in the context of the present study.

Indeed, CFC analyses did not demonstrate significant pre- to post-tACS changes after false-discovery-rate correction, although localized trends were observed in the uncorrected analyses. Therefore, the current electrophysiological findings do not provide sufficient evidence to conclude that tACS restored abnormal CFC or selectively altered cross-frequency phase dynamics. Likewise, because ΔTAR was not associated with cognitive outcomes, the observed cognitive improvements cannot be mechanistically linked to the measured EEG changes.

Several factors may account for the discrepancy between the clinical and cognitive improvements and the absence of robust group-level electrophysiological changes. First, scalp EEG may have limited sensitivity for detecting subtle or spatially distributed alterations in network-level oscillatory coupling. Effects occurring within deeper structures or distributed neural networks may not be adequately captured by conventional scalp recordings. Second, behavioral changes developing over repeated sessions may occur over a different temporal scale from acute electrophysiological changes measured immediately before and after stimulation. Such changes may reflect a combination of longer-lasting stimulation-related effects, repeated exposure, practice, learning, expectancy, or other time-related processes rather than acute session-specific modulation detectable by CFC analysis. Consistent with this possibility, repeated-measures analyses showed significant session effects across DST-F, DST-B, and SDMT, whereas a significant pre- to post-stimulation main effect was observed only for DST-B, was marginal for DST-F, and was absent for SDMT, suggesting that most of the behavioral improvement accrued gradually across sessions rather than acutely within them. Third, substantial inter-individual variability in oscillatory dynamics may have reduced the likelihood of detecting a consistent group-level electrophysiological response.

An additional important limitation is the small sample available for electrophysiological analysis. Only 10 of the 21 participants were included in the final CFC analysis because EEG data from the remaining 11 participants were excluded owing to excessive noise. This substantial reduction in sample size markedly limited statistical power and may have contributed to the absence of significant group-level CFC effects. Accordingly, the observed association between ΔTAR and ΔVAS-L should be regarded as preliminary evidence of a possible relationship between spectral EEG modulation and perceptual tinnitus change rather than evidence of a causal or acute neurophysiological mechanism.

Despite these limitations, the observed cognitive improvements are potentially clinically relevant and warrant further investigation of tACS in patients with tinnitus. Tinnitus is not solely an auditory perceptual disorder but is frequently accompanied by difficulties in attention, working memory, executive function, and cognitive processing. Current tinnitus management strategies, including sound therapy and counseling, primarily target tinnitus perception, distress, or coping and do not directly address associated cognitive difficulties. The improvements observed across several cognitive measures in the present study therefore raise the possibility that neuromodulatory approaches such as tACS may eventually serve as adjunctive interventions targeting both tinnitus-related symptoms and associated cognitive dysfunction. Nevertheless, the present findings should be regarded as hypothesis-generating rather than as evidence of established therapeutic efficacy. The noninvasive nature and practical feasibility of tACS make it an attractive candidate for further evaluation in controlled clinical settings.

Several limitations should be considered when interpreting these findings. Most importantly, the study did not include a sham stimulation group. Consequently, stimulation-related effects cannot be separated from practice, learning, expectancy, placebo effects, spontaneous symptom fluctuation, or other time-related factors. This limitation is particularly relevant to the cognitive outcomes, for which repeated assessment itself can lead to improved performance. In addition, years of education were not recorded. Because educational attainment can influence performance on cognitive measures such as the MoCA, the absence of this information further limits interpretation of the cognitive findings. The lack of an age- and sex-matched healthy control group also prevented direct comparison of baseline resting-state EEG characteristics with normative values.

The modest overall sample size further limits the generalizability and precision of the clinical findings, whereas the substantially smaller EEG subgroup restricts mechanistic interpretation. The exclusion of 11 participants from EEG analyses because of excessive noise markedly reduced the statistical power to detect electrophysiological effects and brain–behavior relationships. Furthermore, the durability of the observed tinnitus-related and cognitive improvements was not assessed beyond the immediate intervention period, and therefore the persistence of these changes remains unknown. Finally, although stimulation frequency was individualized according to each participant’s IAF, other stimulation parameters, including intensity and duration, were fixed across participants. Further personalization of stimulation parameters according to individual neurophysiological characteristics may improve the consistency and magnitude of future responses.

Future studies should therefore employ larger samples and randomized, sham-controlled, longitudinal designs to determine whether the observed clinical and cognitive changes are specifically attributable to CFC-based tACS. Inclusion of age-, sex-, and education-matched comparison groups would facilitate interpretation of baseline cognitive and electrophysiological characteristics. Longer follow-up periods will also be necessary to determine whether any observed benefits persist after stimulation has ended. From a mechanistic perspective, combining high-quality EEG with complementary neuroimaging approaches such as magnetoencephalography or functional magnetic resonance imaging may provide greater sensitivity for identifying spatially distributed or deeper network-level effects. Prespecified analyses linking electrophysiological changes to individual clinical and cognitive responses will be particularly important for determining whether changes in oscillatory activity mediate therapeutic effects. Further optimization and individualization of stimulation parameters based on each patient’s oscillatory characteristics may also improve clinical efficacy.

In summary, repeated sessions of individualized CFC-based tACS were associated with reductions in tinnitus-related functional impact, although subjective ratings of tinnitus loudness and annoyance did not show significant group-level change, and with improvements across several measures of cognitive performance in patients with chronic tinnitus. Cognitive performance improved across repeated sessions, with DST-B additionally showing a significant overall pre- to post-stimulation effect, although no cognitive measure demonstrated a significant tACS × session interaction. Electrophysiologically, robust group-level changes in CFC were not detected after correction for multiple comparisons, whereas an exploratory association between ΔTAR and change in subjective tinnitus loudness provided preliminary evidence of a possible relationship between spectral EEG modulation and perceptual symptom change. Given the absence of a sham control group, the modest sample size, and the limited EEG subgroup, these observations should be regarded as preliminary proof-of-concept findings rather than evidence of a causal therapeutic or neurophysiological effect. Larger sham-controlled studies with longitudinal follow-up and more comprehensive electrophysiological assessment are required to determine the efficacy, durability, and underlying mechanisms of individualized CFC-based tACS for tinnitus.

## Data Availability

The raw data supporting the conclusions of this article will be made available by the authors, without undue reservation.
