# Supplementary material for: Effects of individualized phase-amplitude-coupled transcranial alternating current stimulation on tinnitus and cognition
Source: Front Neurosci. 2026 Sep 30;20:1905125. doi: 10.3389/fnins.2026.1905125 (PMC13662099; doi:10.3389/fnins.2026.1905125)
Supplement: Supplementary file 1 [file Data_Sheet_1.pdf]

Table 1. Simulated Electric-Field Intensities Across Target ROIs

Simulation Condition	Target ROI	Peak E-field (V/m)	99th Percentile (V/m)	Mean E-field (V/m)
**F4 – T7**	**Right DLPFC (F4)**	0.4814	**0.4003**	0.2075
	**Left Auditory (T7)**	0.5395	**0.4692**	0.2052
	Right Auditory (T8)	0.3423	0.3034	0.1598
**F4 – T8**	**Right DLPFC (F4)**	0.4313	**0.3717**	0.1812
	Left Auditory (T7)	0.1330	0.1158	0.0583
	**Right Auditory (T8)**	0.6534	**0.5790**	0.2625

Note: Bold text indicates primary target regions for each montage. ROIs were defined as 20-mm-radius spheres centered on the cortical projections of the respective 10–10 EEG coordinates.

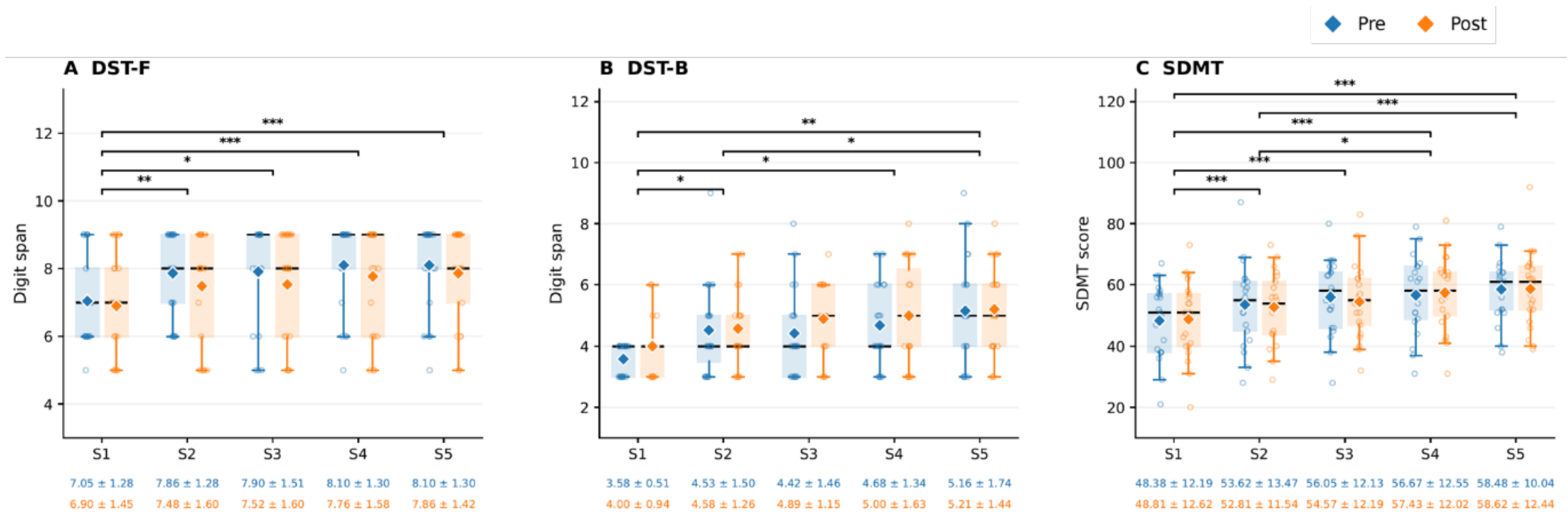

Figure 1. Session-by-session changes in working memory and processing speed measures across five tACS sessions (S1–S5). Box plots depict the median, interquartile range, and full range of individual participant scores (open circles) for pre-tACS (blue diamonds) and post-tACS (orange diamonds) measurements at each session, with group mean ± SD values reported below each session (blue: pre-tACS; orange: post-tACS). (A) Forward Digit Span Test (DST-F) scores showed a significant main effect of session ($F(2.564, 51.278) = 15.498$, $p < 0.001$), with Bonferroni-corrected post hoc comparisons revealing significant improvements between S1 and S2 ($p = 0.002$), S1 and S3 ($p = 0.016$), S1 and S4 ($p < 0.001$), and S1 and S5 ($p < 0.001$). (B) Backward Digit Span Test (DST-B) scores showed a significant main effect of session ($F(2.839, 51.094) = 9.710$, $p < 0.001$) and a significant main effect of tACS (pre- vs post-stimulation; $F(1, 18) = 6.429$, $p = 0.021$), with significant post hoc improvements between S1 and S2 ($p = 0.025$), S1 and S4 ($p = 0.021$), S1 and S5 ($p = 0.002$), and S2 and S5 (p = 0.033). (C) Symbol Digit Modalities Test (SDMT) scores showed a significant main effect of session ($F(3.308, 66.153) = 28.245$, $p < 0.001$), with significant post hoc improvements between S1 and S2, S1 and S3, S1 and S4, and S1 and S5 (all $p < 0.001$), S2 and S4 ($p = 0.024$), and S2 and S5 ($p < 0.001$). Asterisks indicate statistical significance of post-hoc pairwise comparisons ($*p < 0.05$, $**p < 0.01$, $***p < 0.001$).

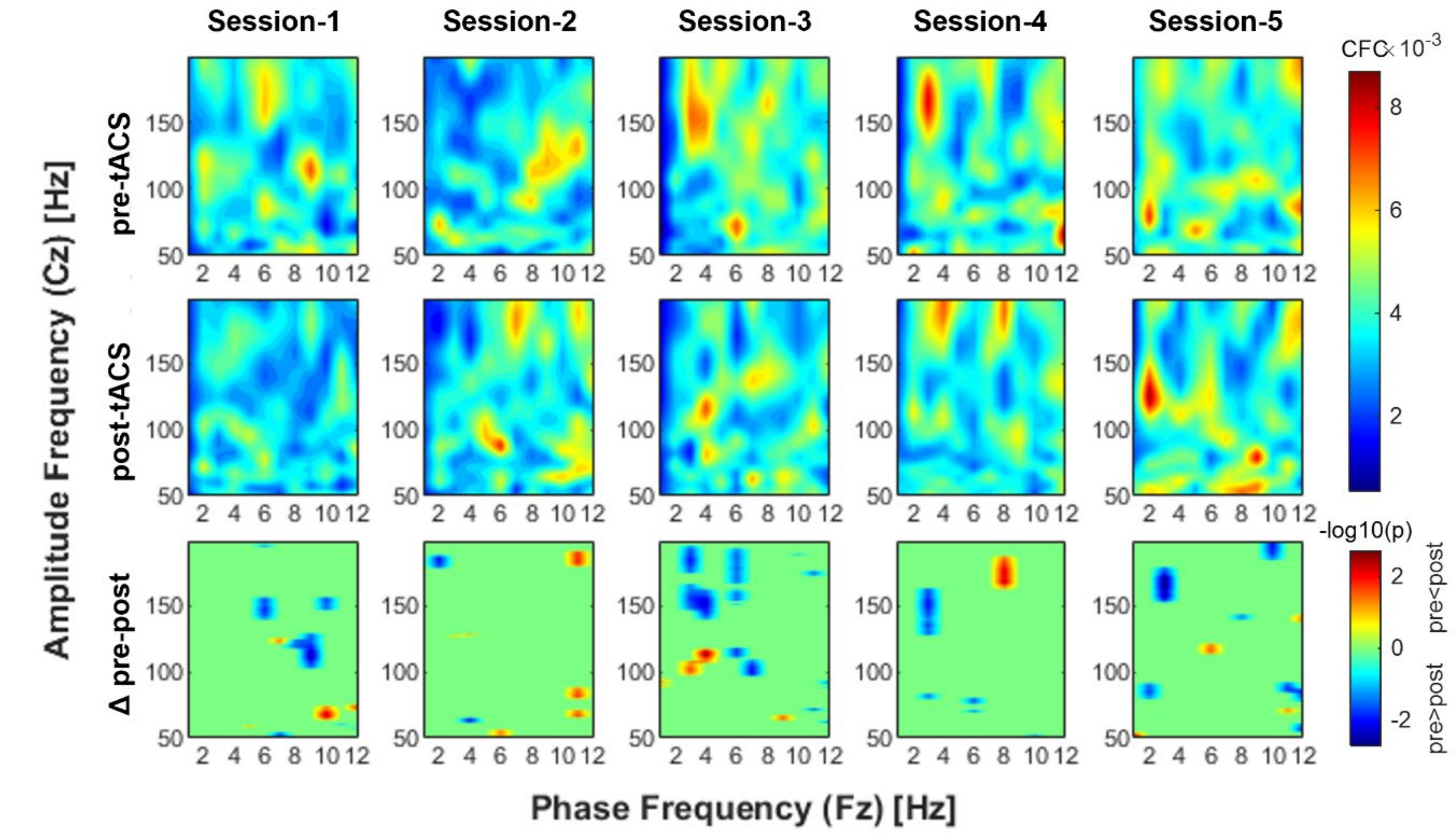

Figure 2. Session-specific comodulograms of cross-frequency coupling (CFC) between phase at Fz and amplitude at Cz in the eyes-open condition. Top two rows: CFC comodulograms at electrode Cz are shown for each session (Sessions 1–5) separately for pre-tACS (top row) and post-tACS (middle row). The color scale represents the strength of phase–amplitude coupling (CFC × 10^{-3}) between phase frequencies (x-axis, electrode Fz) and amplitude frequencies (y-axis, electrode Cz). Bottom row: Uncorrected –log10(p) maps comparing pre- and post-tACS conditions. Warm colors (red) indicate higher post-tACS relative to pre-tACS values, whereas cool colors (blue) indicate higher pre-tACS relative to post-tACS values.

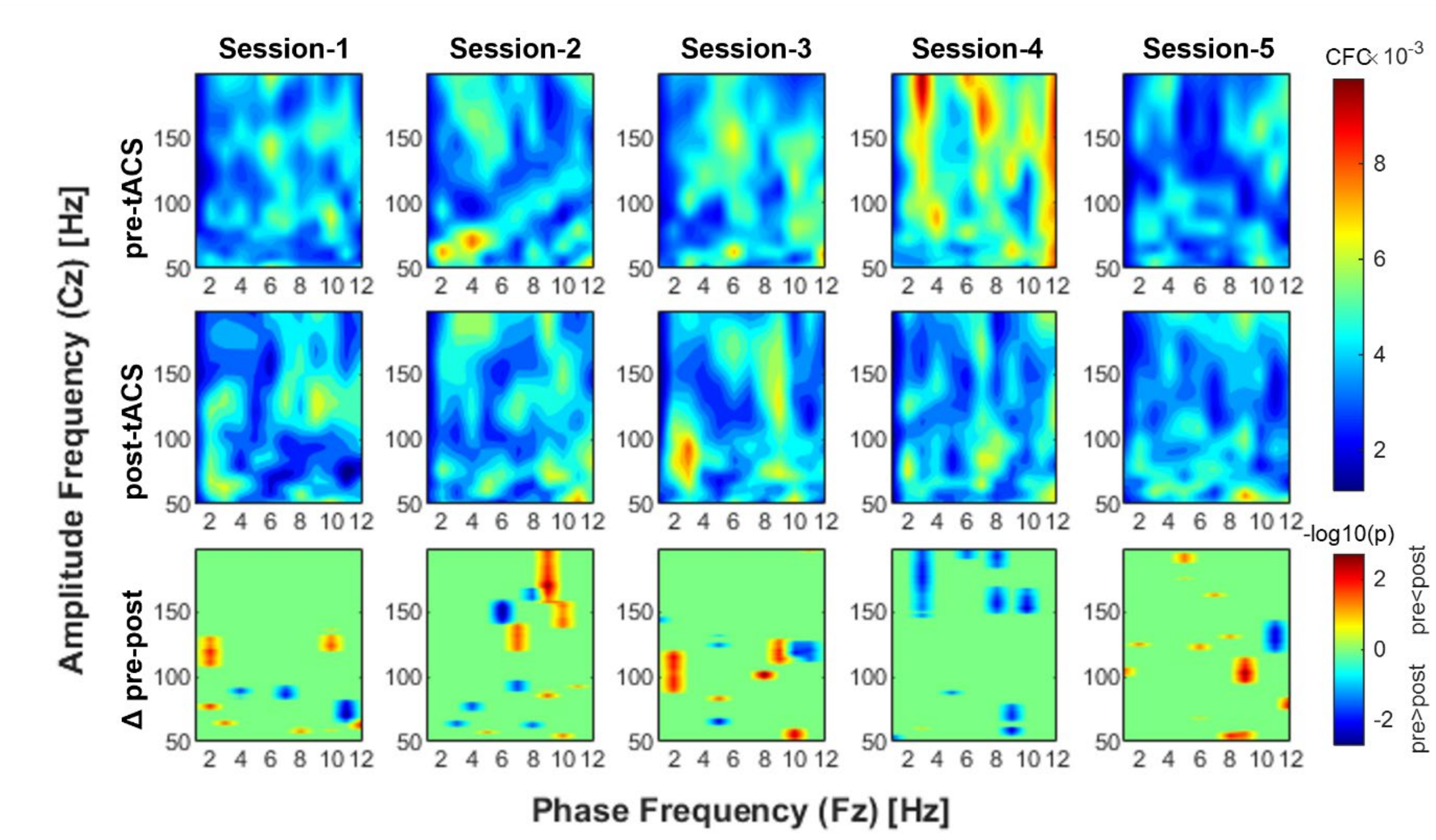

Figure 3. Cross-frequency coupling (CFC) in the eyes-closed condition across pre- and post-tACS sessions. Top row: CFC comodulograms for pre-tACS (Sessions 1–5). Middle row: CFC comodulograms for post-tACS (Sessions 1–5). Bottom row: Uncorrected –log10(p) maps from the comparison between pre-tACS and post-tACS conditions. The x-axis represents phase frequencies (Fz), and the y-axis represents amplitude frequencies (Cz). The color scale indicates the strength of phase–amplitude coupling (CFC × 10^{-3}) for the comodulograms (top and middle rows), and uncorrected –log10(p) values for statistical contrasts (bottom row). Warm colors (red) indicate higher values in the post-tACS condition, whereas cool colors (blue) indicate higher values in the pre-tACS condition. Following FDR correction for multiple comparisons, no significant differences remained (FDR-corrected $p > 0.05$).
