# Supplementary material for: Effects of individualized phase-amplitude-coupled transcranial alternating current stimulation on tinnitus and cognition
Source: Front Neurosci. 2026 Sep 30;20:1905125. doi: 10.3389/fnins.2026.1905125 (PMC13662099; doi:10.3389/fnins.2026.1905125)
Supplement: Supplementary file 2 [file Data_Sheet_2.pdf]

Test Battery

THI and VAS

All patients completed a tinnitus questionnaire that included the Korean version of the Tinnitus Handicap Inventory (K-THI), translated from the original THI developed by the American Tinnitus Association [1], and a visual analog scale (VAS) ranging from 0 to 10 (0 = not annoyed; 10 = extremely annoyed).

Montreal Cognitive Assessment (MoCA)

The Korean version of the MoCA developed by Lee et al. [2] based on the original instrument developed by Nasreddine et al. [3] was used in this study. The MoCA comprises tasks assessing visuospatial skills, executive function, naming, memory, attention, language, abstraction, and orientation. Scores range from 0 to 30, with a cutoff score of 22 considered within the normal range based on Korean normative data reported by Lee et al. (2008). In this study, the MoCA was administered twice: before the first tACS session (S1 pre-tACS) and after the fifth tACS session (S5 post-tACS).

Trail Making Tests (TMT)

The TMT assesses cognitive flexibility, processing speed, sequential processing, divided attention, and visuomotor skills [4,5]. The conventional TMT consists of two parts, TMT-A and TMT-B. TMT-A comprises numbers enclosed in circles, ranging from 1 to 25. Participants are instructed to connect the numbers sequentially in ascending numerical order.

In this study, we used the Korean alphabetical version of the TMT developed by Jang et al. [6]. In this version, participants connect numbers and Korean letters in alternating sequence. The task begins by connecting the first number ('1') to the first letter ('Ka'), followed by the first letter ('Ka') to the second number ('2'), and continues in this alternating manner. As with the MoCA, the TMT was administered twice: before the first tACS session and after the fifth tACS session.

Digit Span Test (DST)

The DST assesses working memory and comprises two subtests: forward digit span (DST-F) and backward digit span (DST-B). In both subtests, participants are presented with digit sequences beginning with three digits and progressively increasing to a maximum of nine digits. Each sequence length includes two trials, yielding a total of 14 trials. In DST-F, participants repeat the digits in the same order in which they were presented, whereas in DST-B they repeat the sequence in reverse order. Testing begins at the easiest level (three digits), and participants advance to the next level after a successful response at the preceding level. The task is

discontinued after two consecutive incorrect responses. The DST score is defined as the highest digit-span level successfully completed. The Korean version of the DST was used in this study. The DST was administered 10 times in total, once before and once after each of the five tACS sessions.

Symbol Digit Modalities Test (SDMT)

The SDMT assesses attention, visual scanning, and processing speed [7]. In the written SDMT, nine symbols are each paired with a digit from 1 to 9. Participants enter the corresponding digit beneath each symbol as quickly and accurately as possible within 90 s. The score is the total number of correct responses. To reduce potential learning effects, four different test forms were randomly selected across administrations. The SDMT was administered 10 times in total, once before and once after each of the five tACS sessions.

References

1. Newman CW, Jacobson GP, Spitzer JBJAoOH, Surgery N. Development of the tinnitus handicap inventory. 1996;122(2)143-8.
2. Lee J-Y, Lee DW, Cho S-J, Na DL, Jeon HJ, Kim S-K, et al. Brief screening for mild cognitive impairment in elderly outpatient clinic: validation of the Korean version of the Montreal Cognitive Assessment. 2008;21(2)104-10.
3. Nasreddine ZS, Phillips NA, Bédirian V, Charbonneau S, Whitehead V, Collin I, et al. The Montreal Cognitive Assessment, MoCA: a brief screening tool for mild cognitive impairment. 2005;53(4)695-9.
4. Llinàs-Reglà J, Vilalta-Franch J, López-Pousa S, Calvó-Perxas L, Torrents Rodas D, Garre-Olmo JJA. The Trail Making Test: Association with other neuropsychological measures and normative values for adults aged 55 years and older from a Spanish-speaking population-based sample. 2017;24(2)183-96.
5. Bowie CR, Harvey PDJNp. Administration and interpretation of the Trail Making Test. 2006;1(5)2277-81.
6. Jang J-W, Kim K, Baek MJ, Kim SJD, Disorders N. A comparison of five types of trail making test in Korean elderly. 2016;15(4)135-41.
7. Ebaid D, Crewther SG, MacCalman K, Brown A, Crewther DPJFian. Cognitive processing speed across the lifespan: beyond the influence of motor speed. 2017;962.
